# Interfacially-Located Nanoparticles Anticipate the Onset of Co-Continuity in Immiscible Polymer Blends

**DOI:** 10.3390/polym9090393

**Published:** 2017-08-25

**Authors:** Martina Salzano de Luna, Andrea Causa, Giovanni Filippone

**Affiliations:** 1 Institute of Polymers, Composite and Biomaterials, National Research Council, P.le Fermi, 80055 Portici, Italy; martina.salzanodeluna@unina.it; 2 Dipartimento di Ingegneria Chimica, dei Materiali e della Produzione Industriale (INSTM Consortium—UdR Naples), University of Naples Federico II, P.le Tecchio 80, 80125 Naples, Italy; andrea.causa@unina.it

**Keywords:** immiscible polymer blends, nanoparticles, morphology, co-continuity

## Abstract

The addition of nanoparticles has recently emerged as a clever tool to manipulate the microstructure and, through it, the macroscopic properties of immiscible polymer blends. Despite the huge number of studies in this field, the underlying mechanisms of most of the nanoparticle-induced effects on the blend microstructure remain poorly understood. Among others, the origin of effect of nanoparticles on the transition from distributed (drop-in-matrix) to co-continuous morphology is still controversial. Here we address this issue through a systematic study on a model blend of polystyrene (PS) and poly(methyl methacrylate) (PMMA) filled with small amounts of nanoparticles (organo-modified clay) selectively located at the polymer–polymer interface. Extraction experiments with selective solvents prove that the nanoparticles significantly anticipate the onset of co-continuity with respect to the unfilled blend. Morphological analyses reveal that such an effect is a consequence of the interconnection of nanoparticle-coated polymer domains. Such “ginger-like” clusters get into contact at low content due to their irregular shape, thus anticipating the onset of co-continuity.

## 1. Introduction

Blending polymers represents an inexpensive route to develop materials with enhanced performances or with new combinations of properties. Due to a generally low mixing entropy, polymers are usually immiscible. As a consequence, the polymer blends are multiphase systems whose properties are dictated, apart from the single constituents, by the small-scale arrangement of the phases generated during mixing [1]. Understanding how to manipulate the morphology of this class of materials at the micron-scale is, hence, highly desirable.

Among the various morphologies possibly resulting from melt mixing routes, co-continuous microstructures have always attracted considerable interest. The main reason is that the mutual interpenetration of the phases typical of this kind of morphology can result in a synergistic combination of the properties of the single constituents [2]. In addition, co-continuous blends can be used as the precursor of polymeric scaffold with controlled porosity, which can be obtained by removing one of the phases with a selective solvent [3,4]. Unfortunately, co-continuous blends are inherently unstable and tend to quickly evolve towards distributed morphologies characterized by lower interfacial area. One strategy to promote and preserve fine co-continuity microstructures envisages the use of block or graft copolymers as compatibilizing agents [5,6]. However, the need of chemically tailoring each compatibilizer to the specific pair of polymers makes such an approach unaffordable in many practical applications. For this reason, great efforts have been made over the last years to identify simple and more general routes for promoting stable co-continuous morphologies. Gubbels et al. [7] and Steinmann et al. [8] were among the first to notice that the range of full co-continuity in polymer blends gets broader in presence of nano-sized fillers. Since then, many researchers have exploited the technological implications of the phenomenon of nanoparticle-induced co-continuity. Among others, Zhang et al. simultaneously improved the stiffness and toughness of nanocomposite blends by promoting the formation of an elastomeric framework upon addition of nanoparticles [9,10]. Filippone et al. preserved the structural integrity of a blend sample at 75 wt % of high density polyethylene well above its melting temperature by blending it with polyamide 6 and inducing the continuity of that phase by means of nanoclay [11]. Extending such an approach to bio-based polymers, Nuzzo et al. enhanced the high-temperature mechanical performances of polylactic acid by developing a blend in which the heat-resistant polyamide 11 represents the minor, and yet continuous, phase [12].

Despite the notable results obtained, the inherent complexity of the previous systems prevents a deep understanding of the mechanisms by which nanofillers promote co-continuity. The need for systematic studies on systems as simple as possible is evident. In addition, the use of reliable experimental techniques is mandatory to avoid misinterpretation of the results. In line with these criteria, we have selected a blend of polystyrene (PS) and poly(methyl methacrylate) (PMMA) filled with small amounts of an organically-modified clay (Cloisite^®^ 15A), which we know from previous studies to be inclined to accumulate at the polymer–polymer interface [13,14]. The effect of the filler on the transition from drop-in-matrix to co-continuous morphology has been investigated through quantitative extraction experiments on unfilled and clay-filled blends at different compositions. Experimental artifacts due to sample size effects have been properly taken into account according to a specific procedure proposed by Galloway et al. [15]. Our results prove that the nanoparticles do really anticipate the formation of co-continuous morphology with respect to the unfilled system. In addition, morphological analyses shed light on the mechanism behind the morphology transition, which appears to be a consequence of a clustering phenomenon between nanoparticle-coated domains of the minor polymer phase.

## 2. Experimental

### 2.1. Materials

The polymeric constituents of the blends are PS (Edistir^®^ 2982 by Polimeri Europa, Ravenna, Italy), with glass transition temperature *T_g_* = 100 °C and zero-shear rate viscosity η_0_ = 3 × 10^3^ Pa·s at *T* = 190 °C, and PMMA (Optix^®^ CA-51 by Plaskolite, Inc., Columbus, OH, USA), having *T_g_* = 110 °C and η_0_ = 4 × 104 Pa·s at *T* = 190 °C. The filler is a montmorillonite modified with dimethyl dihydrogenated tallow quaternary ammonium salt (Cloisite^®^ 15A by Southern Clay Products, Inc., Gonzales, TX, USA) with an organic content of ~43%. The interfacial localization of the selected nanoparticles in the PS/PMMA blends was predicted on the basis of wettability calculations, and already ascertained by means of microscopy observations in recent studies by our group [13,14].

### 2.2. Preparation of the Polymer Blends

PS/PMMA blends at different compositions were prepared by melt compounding using a co-rotating twin-screw extruder (DSM Xplore). The clay/PMMA weight ratio was kept equal to 0.02 for each filled sample. The extrusions were performed at *T* = 190 °C in dry nitrogen atmosphere. The screw speed was set to 150 rpm, corresponding to average shear rates of ~75 s^−1^, and the residence time, accurately controlled by means of a backflow channel, was 240 s. The extrudate was granulated, dried again, and compression molded in the form of disks (diameter 25 mm; thickness 1.5, 3 or 4.5 mm) for subsequent analyses.

### 2.3. Characterization

The extent of continuity of the PMMA phase, φ_PMMA_, in the unfilled and filled PS/PMMA blends was estimated through quantitative extraction experiments. Formic acid (purity grade > 95 wt %, purchased from Sigma Aldrich, St. Louis, MO, USA), which is a selective solvent for PMMA, was used for this purpose. For each blend composition and disk thickness, extraction tests were performed on three different samples. At fixed time intervals, the samples were dried under vacuum at *T* = 70 °C to purge the solvent and then weighed. The solvent extraction procedure was carried out until a weight loss of less than 2% was reached. All the samples remained self-supporting at the end of the experiments.

The morphology of the samples was inspected through scanning (SEM, QUANTA 200F FEG-ESEM by FEI, Hillsboro, OR, USA) and transmission (TEM, Tecnai G2 Spirit Twin T-12 by FEI) electron microscopy. The samples for SEM analyses were gold-sputtered before observation, and the specimens for the analysis of the bulk microstructure were cryo-fractured using liquid nitrogen. The samples for TEM analyses were thin slices (thickness ~100 nm) cut at room temperature using a Leica EM UC7 ultra-microtome equipped with a diamond knife. The morphology of the blends was quantitatively described by referring to the characteristic size and shape of the PMMA phase, evaluated by image analysis of SEM micrographs. The fingerprints of the PMMA domains remaining after immersion in formic acid were manually detected to obtain binary images, which were subsequently analyzed by means of an open source image processing software (ImageJ). The equivalent radius of the *i*-th domain, *R_eq,i_*, was evaluated as

(1) $$R_{eq,i}=\sqrt{\frac{A_{i}}{\pi }}$$

where *A_i_* corresponds to the measured domain area amplified by a factor of 4/π in order to account for the systematic underestimation of the dimensions of an object from its print on a flat surface [16]. To assess the effect of the filler on the shape of the polymeric phases, the circularity of the *i*-th domain, *C_i_*, was evaluated as

(2) $$C_{i}=\frac{4\pi A_{i}}{P_{i}^{2}}$$

where *P_i_* is the perimeter of the domain.

## 3. Results and Discussion

### 3.1. Effect of Nanoparticles on the Co-Continuity of Immiscible Polymer Blends

**Figure 1:**
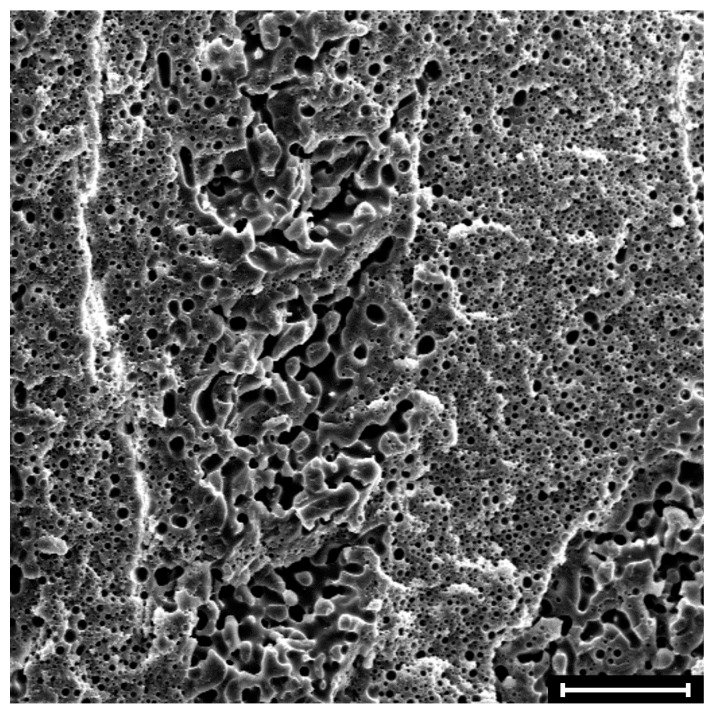
SEM micrograph showing the morphology of an unfilled PS/PMMA sample at Φ_PMMA_ = 0.35 (disk-shaped specimen; diameter *d* = 25 mm, height *h* = 4.5 mm). The degree of continuity of the PMMA phase in this sample is φ_PMMA_ ≈ 0.28. The sample surface was etched with formic acid, which selectively removes the PMMA phase. The scale bar corresponds to 100 μm.

A well-established technique for the detection of co-continuity in an immiscible polymer blend is the selective removal of either of the phases from the sample using an appropriate solvent [17]. In quantitative extraction experiments, a sample is immersed in a selective solvent and the changes in mass during the extraction process of a phase “*i*” are monitored over time. The degree of continuity of the soluble phase “*i*” is measured as

(3) $$\varphi_{i}=\frac{m_{i,0}-m_{i,f}}{m_{i,0}}$$

where *m_i,_*_0_ and *m_i,_*_f_ represent the nominal masses of “*i*” inside the sample at the beginning and at the end of the extraction test, respectively. The value of φ*_i_* is a function the weight fraction of “*i*” in the blend, Φ*_i_*, and it ranges from 0 to 1 when the morphology passes from a distributed microstructure, made of isolated domains of “*i*” suspended in the matrix of the second phase, to a co-continuous one, in which both phases are continuous in the space [18]. Intermediate values of φ*_i_* indicate the coexistence of dispersed and continuous domains of “*i*”. An example of such a composite morphology is shown in Figure 1, where the SEM picture of the unfilled blend at PMMA weight fraction Φ_PMMA_ = 0.35 is reported. The compresence of micron-sized drops embedded in the PS matrix and larger, irregularly-shaped domains interpenetrated with the PS phase can be clearly noticed. Quantitative extraction experiments carried out on this sample (disk-shaped; diameter *d* = 25 mm, thickness *h* = 4.5 mm) returned φ_PMMA_ ≈ 0.28.

**Figure 2:**
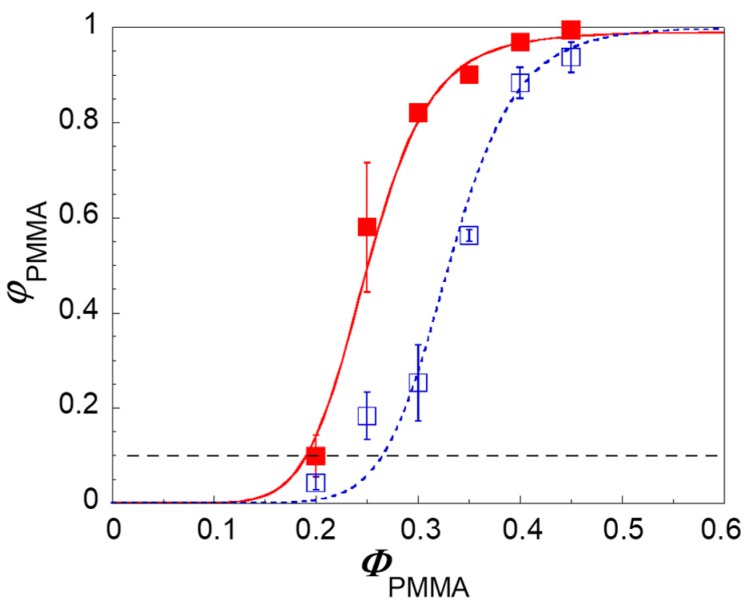
Degree of continuity of PMMA as a function of the PMMA content for unfilled (empty symbols) and clay-filled (full symbols) PS/PMMA blends. The data are average values obtained from three independent tests and refer to disk-shaped samples (*d* = 25 mm, *h* = 1.5 mm). The error bars are the standard deviations. Lines are a guide for the eye.

The addition of nanoparticles is well-known to radically alter the phase morphology of immiscible blends [2]. In general, the filler causes a significant reduction of the size of the phases, but some studies also reported an effect on the onset of co-continuity, Φ_onset_, defined as the composition of the blend at which the dispersed phase starts to interconnect [19,20,21,22,23]. The degree of continuity of the PMMA phase is shown in Figure 2 as a function of the amount of PMMA for unfilled and clay-filled samples.

The data in Figure 2 clearly indicate that interfacially-located nanoparticles anticipate the content of PMMA in the blend at which φ_PMMA_ starts to increase, Φ_onset_. Conventionally defining the latter as the content of PMMA at which φ_PMMA_ = 0.1, the effect of the nanoparticles can be quantified in a reduction of Φ_onset_ of about 10%.

**Figure 3:**
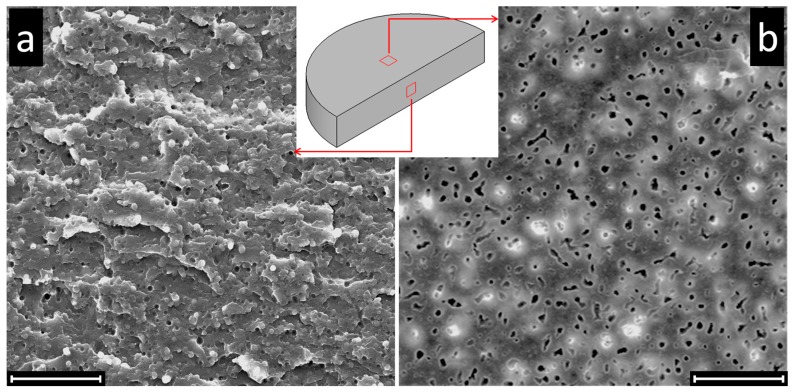
SEM micrographs showing (**a**) the bulk and (**b**) the surface of a clay-filled PS/PMMA sample at Φ_PMMA_ = 0.20 after selective extraction tests in formic acid (*h* = 4.5 mm). Scale bars are 50 μm.

It is important to observe that the φ_PMMA_ values measured through quantitative extraction experiments are overestimated due to the so-called “sample size effect” [15]. This systematic error is due to the removal of a fraction of isolated PMMA domains contained in a surface layer of thickness *δ* during extraction tests, which artificially increases the measured value of φ_PMMA_. This effect can be clearly seen in Figure 3, where SEM micrographs of bulk and surface of a disk-shaped sample at Φ_PMMA_ = 0.20 are reported at the end of quantitative extraction experiments.

At this composition, the sample exhibits drop-in-matrix morphology. Consequently, the PMMA droplets in the bulk cannot be reached by the solvent, as clearly visible from the micrograph of Figure 3a. Nevertheless, a non-zero value PMMA phase continuity is measured (φ_PMMA_ ≈ 0.10) due to the removal of the PMMA isolated domains on the samples surface, which indeed appears full of holes (Figure 3b).

**Figure 4:**
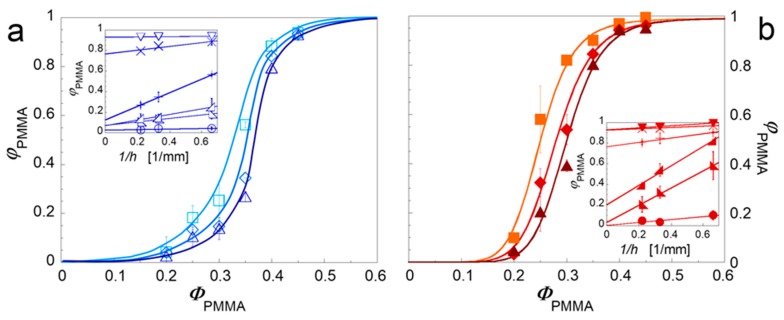
Degree of continuity of PMMA as a function of PMMA weight content for unfilled (**a**) and clay-filled (**b**) PS/PMMA blends for samples of different thickness: 1.5 mm (squares), 3 mm (diamonds), and 4.5 mm (triangles). Lines are a guide for the eye. The insets show the same data as a function of the reciprocal of the sample thickness; these data are fitted according to Equation 4.

In general, the amount of non-continuous and yet removable fraction of polymer “*i*” responsible for the overestimate of φ*_i_* depends on the ratio between surface and volume of the tested sample. Galloway et al. derived a simple procedure to extrapolate the “true” degree of continuity of the soluble phase “*i*”, φ*_i,t_*, by testing samples with same shape but different thickness [15]. Dealing with unfilled blends of poly(ethylene oxide) and polystyrene, the authors showed that the degree of continuity estimated through quantitative extraction tests is significantly overestimated for intermediate values of φ*_i_*. Moreover, the relevance of the size effect when comparing the results of experiments using samples with different sizes and shapes was emphasized. We observe that the morphology of the polymer phases also plays a role, and this can be particularly important in presence of nanoparticles able to greatly alter the morphology of the polymer phases. To investigate this aspect and substantiate the data of Figure 2, quantitative extraction tests were performed on disk-shaped samples having the same diameter but different thicknesses, namely *h* = 1.5, 3, and 4.5 mm according to the procedure by Galloway et al. The following linear relationship is expected:

(4) $$\varphi_{PMMA}=m\left(\frac{1}{h}\right)+\varphi_{PMMA,t}$$

where the slope *m* is related of the depth of solvent penetration δ, being a measure of the relevance of the sample size effect (φ_PMMA_ ≡ φ_PMMA,*t*_ for *m* = 0). The results of the quantitative extraction experiments are shown in Figure 4 as a function of Φ_PMMA_.

**Figure 5:**
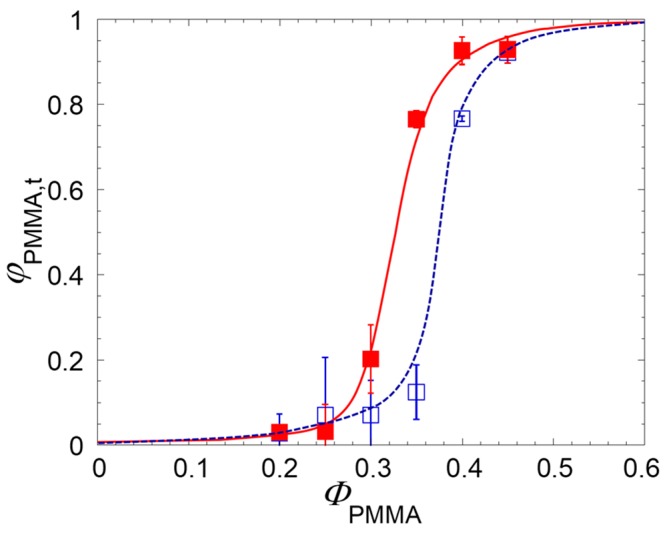
Degree of continuity of the PMMA phase corrected from the sample size effect as a function of PMMA content for unfilled (empty symbols) and clay-filled (full symbols) blends. The lines are a guide for the eye.

As the thickness of the specimen increases, or rather as the surface-to-volume ratio decreases, the experimental Φ_PMMA_ values decrease. The insets shown the data reported in terms of 1/*h*. The intercepts of the fitting lines (Equation (4)) represent the φ_PMMA*,t*_ values, which are reported in Figure 5 for the unfilled and clay-filled systems.

**Figure 6:**
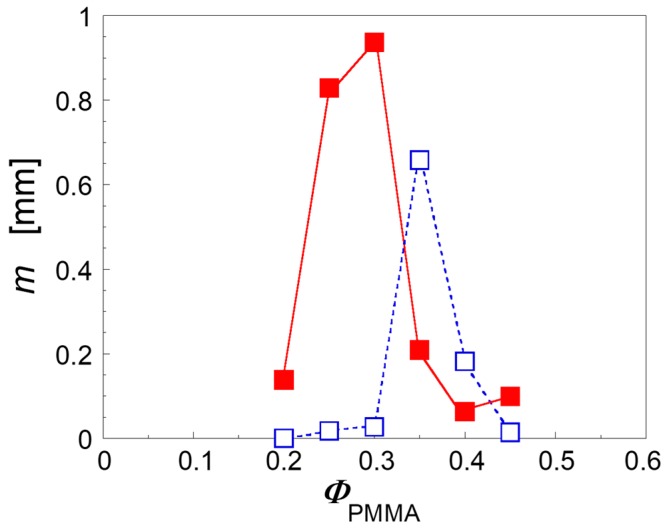
Slopes of the fitting curves reported in the insets Figure 4 as a function of PMMA weight content for unfilled (empty symbols) and clay-filled (full symbols) blends.

The effect of the correction is an increase of the Φ_onset_ values in both the unfilled and clay-filled blends respect to Figure 2. The corrected data, however, confirm that the interfacial plate-like nanoparticles do really anticipate the formation of co-continuous morphology with respect to the unfilled system. It is important to observe that the actual reduction of Φ_onset_ induced by the filler is less pronounced than what emerged in Figure 2 with a single set of quantitative extraction data. As shown in Figure 6, this is a consequence of a greater sample size effect in the clay-filled system.

Higher values of *m* mean thicker superficial layers in which the solvent is able to penetrate removing the isolated polymeric domains responsible for the artificial increase in the Φ_PMMA_ values. In other words, the superficial PMMA domains in the clay-filled system protrude more in depth into the bulk of samples. Aim of the next Section is to shed light through targeted morphological analysis on the physical mechanism underlying this effect and, more in general, on the ability of the nanoparticle to promote phase continuity.

### 3.2. Mechanisms of Nanoparticle-Induced Alteration of the Co-Continuity Interval

**Figure 7:**
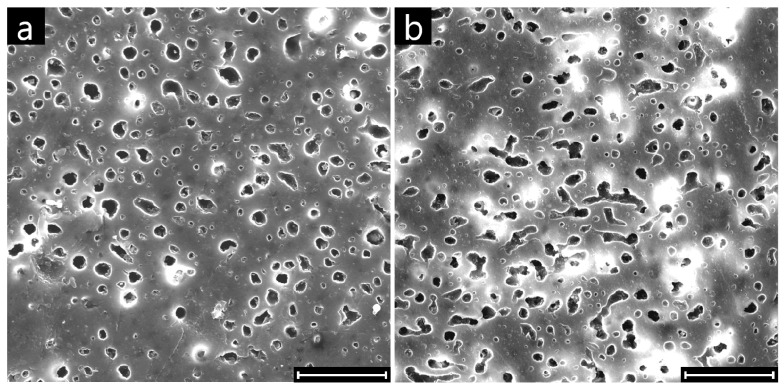
SEM micrographs of the surfaces of (**a**) unfilled and (**b**) clay-filled PS/PMMA samples at Φ_PMMA_ = 0.30. Scale bars are 30 μm.

Representative SEM micrographs of the surface of neat and clay-filled samples at composition close to phase inversion (Φ_PMMA_ = 0.30) are shown in Figure 7.

**Figure 8:**
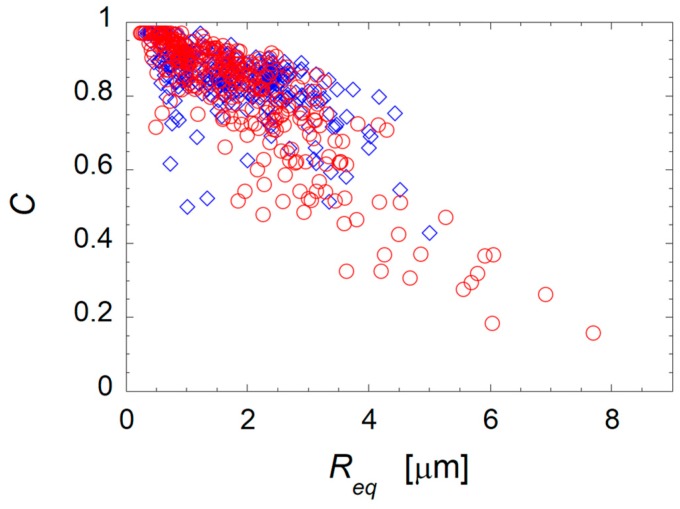
Circularity of each domain of PMMA as a function of its size for the unfilled (diamonds) and clay-filled (circles) PS/PMMA samples at Φ_PMMA_ = 0.30. Note that both datasets contains 300 points.

The PMMA domains extracted from the sample surface appear more irregular in shape in the filled blend than in the unfilled one. To corroborate this observation and quantify the effect of the nanoparticles on the characteristic shape of the PMMA domains, image analysis was carried out on various SEM micrographs of the neat and clay-filled blends at Φ_PMMA_ = 0.30. The results are reported in Figure 8, where the circularity of the superficial PMMA domains is reported as a function of their equivalent radius.

**Figure 9:**
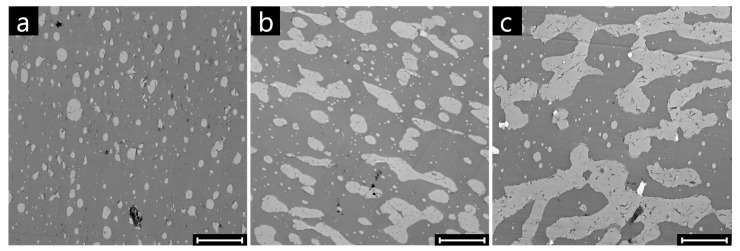
TEM micrographs of clay-filled PS/PMMA samples at Φ_PMMA_ = (**a**) 0.20; (**b**) 0.30; (**c**) 0.45. The bright phase is the PMMA. Scale bars are 5 μm.

Both systems exhibit a considerable portion of relatively small (R_eq_ < 3 μm) and round (0.8 < C < 1.0) domains. On the other hand, the data are more scattered for the filled system, which presents a considerable number of domains with large characteristic size and low circularity. Such big and irregularly-shaped superficial domains are likely to penetrate more in depth into the sample, thus explaining the increase of the solvent penetration depth on the basis of the higher values of *m* found for the clay-filled system. At the same time, the occurrence of irregularly-shaped domains of the dispersed phase in the bulk of the clay-filled samples lowers the percolation threshold of the PMMA phase, likely playing a key role in anticipation of the onset of co-continuity. To shed light on this mechanism, targeted morphological analyses were carried out. The TEM micrographs of selected clay-filled samples at Φ_PMMA_ = 0.20, 0.30, and 0.45 are shown in Figure 9. The samples exhibit φ_PMMA*,t*_ values of about 0.05, 0.20, and 0.90, respectively, revealing the presence of completely dispersed, partially interconnected, and fully continuous PMMA phase.

At the lowest content, the PMMA phase appears in the form of submicron- and micron-sized droplets suspended in the PS matrix (Figure 9a). The presence of non-spheroidal domains can be occasionally noticed. This is due to the bending stiffness of the interfacial nanoparticles, which frustrate the shape relaxation of the polymeric domains and stabilize distorted structures even in cases of partial interfacial coverage [13]. The morphology of the blend at the intermediate composition is more variegated, and isolated PMMA droplets coexist with bigger, irregularly-shaped domains (Figure 9b). The non-negligible value of φ_PMMA*,t*_ ≈ 0.20 indicates that some of these structures are actually interconnected throughout the sample. Finally, the sample at Φ_PMMA_ = 0.45 exhibits the typical morphology of co-continuous blends. Note that the presence of apparently isolated small domains of PMMA is consistent with the value of φ_PMMA*,t*_ ≈ 0.90 found with the quantitative extraction experiments.

**Figure 10:**
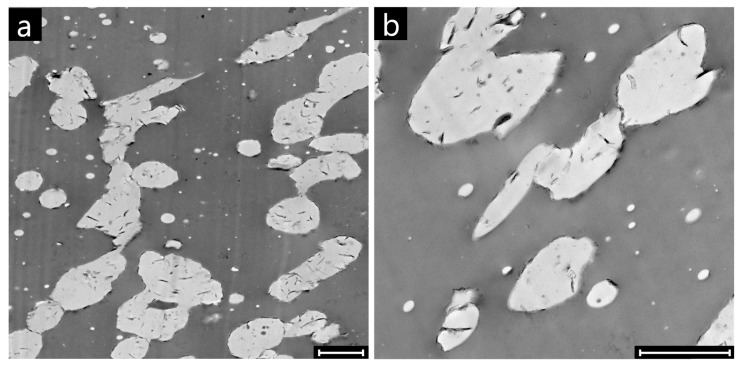
TEM micrographs of clay-filled PS/PMMA samples at Φ_PMMA_ = 0.30. Scale bars are 2 μm. Part (**a**) is readapted from [2], with permission from Elsevier.

The high magnification TEM micrographs of the clay-filled sample at Φ_PMMA_ = 0.30 shown in Figure 10 unveil the physical origin of the incipient interconnection of the PMMA domains. The latter derive from the clustering of distinct PMMA domains, which are clearly recognizable due to the presence of the clay platelets located on their surface. The formation of such “ginger-like” clusters of clay-coated drops is the reason why some nanoparticles are visible inside the PMMA phase in contrast to their thermodynamic propensity to locate at the polymer-polymer interface.

The driving force for drop clustering in the studied systems originates from the strong van der Waals interactions between clay-coated drops. The attractive force between two drops of radius *R* at a distance *h* from each other is approximately given by [24]:

(5) $$F=\frac{A_{iji}R}{12h}$$

where *A_iji_* is the Hamaker constant of two bodies of a medium “*i*” interacting across a medium “*j*”. In the case of clay-coated drops, the value of *A_iji_* is estimated to be two orders of magnitude higher than that of bare drops [13], and stable drop clusters hence form. Compared to the bridging-dewetting mechanism described by Thareja et al. [25], drop clustering is characterized by the fact the pristine domains keep their individuality without merging together and/or relaxing back towards spheroidal shapes. The preservation of the irregular shapes is ensured by the bending stiffness of the interfacial clay particles, which offset the interfacial tension responsible for the shape relaxation processes in unfilled blends. Although other mechanisms cannot be excluded, the reduced percolation threshold of distorted domains [26] is believed to play a role in the lowering of onset of PMMA continuity detected in the studied systems.

## 4. Conclusions

The role of small amounts of interfacially-located clay nanoparticles in promoting the formation of co-continuous morphology in PS/PMMA blends at different compositions was investigated. The degree of continuity of the PMMA phase was assessed through selective extraction experiments. Samples with different surface-to-volume ratios were tested to account for the experimental artifacts due to the removal of isolated superficial domains (“sample size effect”). The analysis showed that the overestimate of the degree of co-continuity due to this systematic error is more pronounced for filled blends. Nonetheless, it is confirmed that the nanoparticles do really anticipate the occurrence of co-continuous morphology in the investigated PS/PMMA system. Morphological analyses revealed that the filler promotes the clustering of the clay-coated PMMA domains. Such irregularly-shaped clusters increase the degree of connectivity of the minor polymer phase, which percolate at lower contents explaining the anticipated onset of co-continuity detected in the filled blends.
